# Characteristics of the blood–brain barrier in pediatric brain tumors

**DOI:** 10.3389/fped.2025.1646641

**Published:** 2025-08-29

**Authors:** Qiang Gao, Dengpan Song, Dingkang Xu, Xinyi Chai, Ming Ge

**Affiliations:** ^1^ Department of Neurosurgery, Beijing Children’s Hospital, National Center for Children’s Health, Capital Medical University, Beijing, China; ^2^ Department of Neurosurgery, The First Affiliated Hospital of Zhengzhou University, Zhengzhou, China; ^3^ Department of Neurosurgery, Guangdong Provincial People’s Hospital (Guangdong Academy of Medical Sciences), Southern Medical University, Guangzhou, China

**Keywords:** pediatric brain tumors, blood-brain barrier, tumor heterogeneity, permeability, drug delivery

## Abstract

The blood–brain barrier (BBB) plays a vital role in maintaining central nervous system homeostasis but poses a major obstacle to effective drug delivery in pediatric brain tumors. BBB integrity varies significantly in pediatric brain tumors compared to adult ones, and is influenced by the tumor type, molecular subtype, and anatomical location. This review discusses the heterogeneous nature of the BBB across various pediatric brain tumors, including low-grade gliomas, diffuse midline gliomas, medulloblastomas, ependymomas and craniopharyngiomas. We review histological, molecular, and imaging evidence to highlight differences in BBB permeability and their implications for therapeutic delivery and treatment resistance. Special consideration is given to advanced drug delivery strategies, such as focused ultrasound and BBB-disrupting agents, which have been tailored to the unique barrier properties of each tumor subtype. A deeper understanding of tumor-specific BBB architecture is essential for tailoring treatment strategies and improving outcomes in pediatric brain cancer.

## Introduction

Pediatric brain tumors are among the most common solid tumors in children and are a leading cause of cancer-related mortality in this population worldwide (1–4). In the United States, central nervous system (CNS) tumors rank the second most common cancer, second only to leukemia, in children aged 0–19 years; however, their mortality rate has surpassed that of leukemia, making CNS tumors the leading cause of cancer-related death in children (3). Approximately 80%–90% of the newly diagnosed pediatric brain tumor cases each year occur in low- and middle-income countries, which together account for approximately 88% of the global pediatric population (5). Studies in China have demonstrated significant regional differences in the annual incidence of pediatric brain tumors (6). Moreover, the peak age for pediatric brain tumors is between 0 and 14 years (7), and some studies have reported a slight male predominance, with a male-to-female ratio of approximately 1.2:1 (8). Common pediatric brain tumor types include low-grade gliomas (accounting for approximately 30% of all gliomas) (9), medulloblastomas (10%–15.2%), ependymomas, craniopharyngiomas, and diffuse midline gliomas, with approximately half of these tumors exhibiting malignant features (10). Anatomically, the most common sites of occurrence are the posterior fossa, ventricular system, and supratentorial regions (10).

The microvasculature of the CNS, collectively termed the blood–brain barrier (BBB), tightly controls the passage of ions, molecules, and cells into the CNS to maintain homeostasis within this evolutionarily conserved region. Drug penetration across the BBB is impacted by factors such as molecular size, lipid solubility, and efflux transporters (11). With respect to the molecular size, most publications report an absolute cutoff of 400–600 Da (12), and the largest substance reported to cross the BBB to date had a molecular weight of 7.8 kDa (13). BBB development occurs synchronously with CNS angiogenesis, and its structure and function support the metabolic demands of neurons and glial cells (14). During development, the brain vasculature acquires a series of specialized molecular and cellular properties, such as tight junction proteins and efflux transporters, which collectively form the BBB and limit passive diffusion between the blood and brain parenchyma (14, 15).

In pediatric brain tumors, the BBB often undergoes structural and functional alterations, forming a blood–brain tumor barrier whose degree of disruption exhibits marked heterogeneity both across different tumor types and among different regions within the same tumor. This heterogeneity likely plays a crucial role in the permeability and therapeutic efficacy of drugs. Moreover, the unique developmental state of the pediatric BBB may further restrict the permeation of therapeutic agents, especially large molecules, such as monoclonal antibodies (mAbs), thereby influencing treatment outcomes (16, 17). To overcome these limitations, recent research efforts have focused on the exploration of strategies such as the use of nanoparticle carriers and BBB-penetrating peptides (e.g., BBB-modulating peptides) to increase drug delivery efficiency; however, these approaches must carefully consider differences in metabolism and sensitivity to toxic substances inherent to the developing pediatric brain (18). Existing BBB models, including stem cell-derived models and microfluidic chip systems, are predominantly based on adult tissue data and lack the capacity to simulate pediatric brain physiology accurately (19). Therefore, there is an urgent need to establish *in vitro* models that more closely mimic the physiological state of the developing brain, enabling deeper investigations into the dynamic changes in the BBB of pediatric brain tumors (20).

A comprehensive understanding of BBB characteristics in pediatric brain tumors is critical for optimizing therapeutic strategies, improving targeted drug delivery efficiency, and ultimately improving patient outcomes. This review focuses on the features of the BBB in several common pediatric brain tumors (21), with the aim of providing a theoretical foundation and research directions for their precise treatment in the future.

## Low-grade gliomas

**Figure 1:**
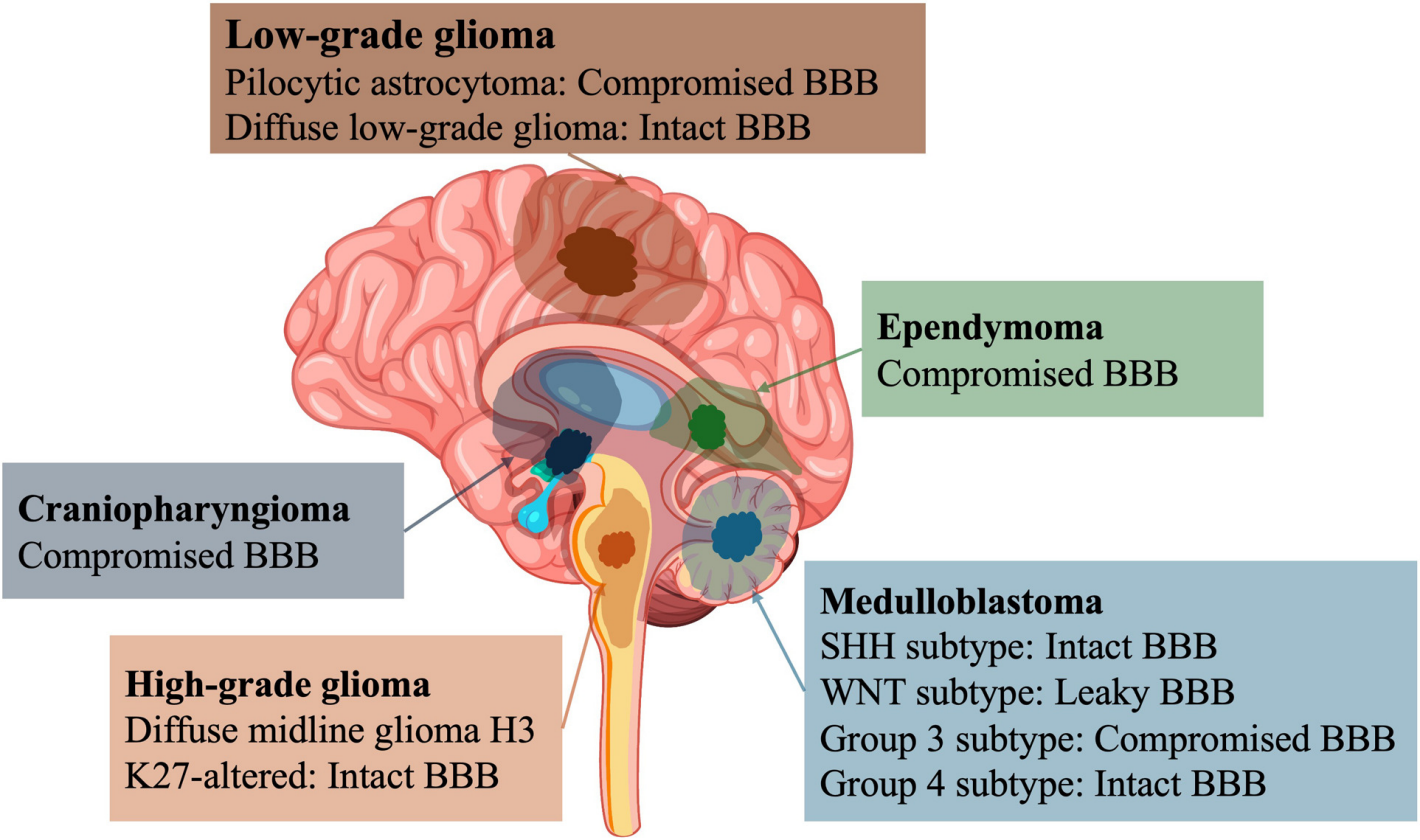
Localization and BBB characteristics in pediatric brain tumors. BBB, blood–brain barrier; SHH, sonic hedgehog; WNT, wingless.

Pediatric low-grade gliomas (pLGGs) and glioneuronal tumors represent the most common brain tumors, accounting for nearly 30% of pediatric CNS neoplasms. pLGGs are defined as grade 1 or 2 per the recent World Health Organization (WHO) 2021 classification (22). Overall, the BBB function in pLGGs remains relatively intact, with tumor cells causing minimal disruption. This BBB preservation has been observed in magnetic resonance imaging (MRI) examinations. Typically, low-grade gliomas do not exhibit marked contrast enhancement, which distinguishes them from high-grade gliomas. The relatively normal vascular architecture of low-grade gliomas in children contributes to the maintenance of BBB integrity (Figure 1). This integrity protects the brain tissue around the tumor from harmful substances that are secreted by tumor cells, limiting the rapid growth and metastasis of the tumor. According to the report by Hong CS et al. (23), an abundant glial fibrillary acidic protein (GFAP) signal was observed in pediatric pilocytic astrocytoma, which was consistent with the astrocytic lineage of the tumor cells. Aquaporin-4 (AQP4), a marker of astrocytic end-foot processes, has been widely used as a reliable indicator of the structural and functional integrity of astrocyte–endothelial cell interactions (24). AQP4 expression was reduced and exhibited a disorganized pattern within the pilocytic astrocytoma tumor tissue, suggesting that the tumor cells were unable to fulfill their physiological role in supporting the BBB, thereby facilitating increased permeability for therapeutic agents to access the lesion from the vasculature. In low-grade diffuse astrocytoma, immunofluorescence staining revealed an intact BBB, which was characterized by strong GFAP and AQP4 signals surrounding the microvasculature. Notably, AQP4 staining was sufficiently intense to delineate the contours of CD31-positive vessels independently (23). On the basis of data from animal models, Tan J et al. suggested that the use of an I6P7 peptide-mediated MRI probe could be a potential strategy for overcoming the BBB integrity and diagnosing low-grade gliomas (25). The integrity of the BBB remains a major obstacle for conventional chemotherapeutic agents to reach the tumor parenchyma. However, focal BBB disruptions may create opportunities for the use of targeted therapies and advanced drug delivery systems. Recently, focused ultrasound (FUS) combined with microbubble technology has been employed as a noninvasive and reversible method to locally open the BBB, thereby enhancing the penetration of antitumor drugs into pLGGs. Preliminary studies have shown promising results (26, 27). In terms of surgical treatment, sodium fluorescein is among the ideal candidates as an intraoperative marker for actual recognition of tumor extension, since it accumulates in areas with an altered blood–brain barrier, a typical characteristic of pediatric gliomas, and has a low rate of adverse events, which greatly facilitates complete surgical resection (28). To better model and understand the complex BBB status in pLGGs, researchers have utilized human induced pluripotent stem cells (hiPSCs) to develop subtype-specific *in vitro* models. These models can reflect the interactions between different molecular subtypes of pLGGs and the BBB, providing new technical avenues for personalized drug screening and preclinical platform development (29, 30), However, they are limited by the absence of immune system components and a lack of long-term functional stability.

## High-grade gliomas

High-grade gliomas account for approximately 10% of CNS tumors in children and are the leading cause of cancer-related death in individuals under 19 years of age. According to the 2021 WHO classification, pediatric diffuse high-grade gliomas are categorized into four distinct subgroups, with diffuse midline glioma (DMG), H3 K27-altered, being the most common subtype (21). DMG is a highly infiltrative and aggressive pediatric brain tumor, with a median survival of approximately nine months following diagnosis. These tumors arise in midline structures of the central nervous system, most commonly in the pons, where they are referred to as diffuse intrinsic pontine gliomas (DIPGs), as well as in the thalamus or spinal cord. More than 80% of DMG cases harbor a specific lysine-to-methionine substitution at position 27 (K27M) in histone H3 variants, occurring either in the canonical H3C1/H3C2 genes (H3.1-K27M) or in the alternative histone H3 variant H3-3A (H3.3-K27M) (31). MRI with a gadolinium-based contrast agent is the most commonly employed method for assessing BBB disruption. Unlike certain CNS tumors that exhibit a compromised BBB, DMG is characterized by a relatively intact BBB (Figure 1) (32–34). However, heterogeneity within the tumor's blood–tumor barrier can result in focal areas of increased permeability. In contrast to many other malignant CNS tumors, the vasculature within DMGs generally retains BBB integrity (35–39). This preservation is largely maintained by tight junction proteins and specialized interendothelial structures that constitute an effective barrier, limiting the penetration of large-molecule therapeutics into the tumor microenvironment. Claudin-3, a key tight junction protein, is known to be overexpressed in ovarian cancer cells, where its upregulation is associated with a reduction in the repressive histone mark H3K27me3 at its promoter region. Similarly, histone mutations that lead to decreased H3K27me3 may contribute to aberrant upregulation of Claudin-3 expression in DMG (40). As a result, the intact BBB presents a major challenge to the delivery and efficacy of molecularly targeted therapies and chemotherapeutic agents (36, 37, 41). Notably, a study by McCully et al. demonstrated that the BBB is not uniformly impermeable; rather, it is heterogeneous, as evidenced by the differential penetration of temozolomide between the brainstem and cortical regions (42). To date, more than 250 DMG-targeting clinical trials have failed to overcome the BBB limitation, resulting in persistently high treatment failure rates (38). At the molecular level, the hallmark genetic alteration in DMG, the H3 K27M mutation, causes global hypomethylation of histone H3, potentially regulating the expression of BBB-related genes through epigenetic mechanisms and thus influencing the dynamic functionality of the barrier (43–45). In an *in vitro* study that investigated the DIPG-specific BBB, Deligne et al. reported that over the course of one week of DIPG cell development, there was no significant change in either the permeability of the barrier to chemotherapeutic agents or the expression levels of efflux transporters (34), which underscores the complex and multifactorial nature of chemoresistance in DMGs (34). Imaging studies using diffusion-weighted imaging (DWI) and apparent diffusion coefficient (ADC) analysis have revealed significant differences in ADC values between H3 K27M-mutant DMGs and other midline gliomas, with these parameters potentially serving as indirect indicators of the BBB functional status and tumor microenvironment characteristics (46, 47). With respect to treatment, small molecules such as ONC201 (dordaviprone) or epigenetic agents demonstrate relatively good BBB penetration and have shown preliminary efficacy in some patients with the H3 K27M mutation, making these agents a current focus of clinical research (48). Several studies have reported the expression of efflux transporters, such as P-glycoprotein/MDR1 (P-gp) and breast cancer resistance protein 1 (BCRP1), not only on the surface of DIPG cells but also within the surrounding tumor microenvironment (49). P-gp, in particular, contributes to reduced intracellular drug accumulation by actively exporting therapeutic agents in an ATP-dependent manner (50). Although CAR-T-cell therapy has shown some potential to penetrate the BBB, many immunotherapeutic agents, including immune checkpoint inhibitors, are poorly delivered to the tumor site (51, 52). Moreover, the tumor immune microenvironment in the DMG is generally immunosuppressive and, in combination with the intact BBB, further diminishes the efficacy of systemic therapies (53). Radiotherapy, as the current standard of care, can temporarily alleviate symptoms but does not effectively overcome BBB-related drug delivery barriers. However, its long-term efficacy and BBB modulation effects remain controversial (54). Various techniques that are aimed at bypassing or disrupting the BBB, such as convection-enhanced delivery (CED) and focused ultrasound, have demonstrated promising results. Clinical trials investigating techniques such as CED and FUS for targeted drug delivery to the brain are increasing in number (55, 56) In addition to enhancing drug penetration, FUS can transiently disrupt the BBB, enabling the release of tumor-derived biomarkers into the bloodstream, which may facilitate noninvasive monitoring of tumor progression and treatment response through liquid biopsy. Furthermore, FUS holds promise for delivering larger therapeutic molecules, such as monoclonal antibodies, to otherwise inaccessible brain regions. These approaches hold significant potential for overcoming the formidable challenge that the BBB presents to drug delivery to the central nervous system (35, 44). Future breakthroughs in DMG treatment may depend on effective modulation of the BBB combined with molecular-targeted and immunotherapeutic strategies.

## Medulloblastoma

Medulloblastoma is one of the most common malignant CNS tumors in children. During tumor progression, the BBB undergoes pathological changes, resulting in a structurally and functionally compromised BBB. The extent of BBB disruption is significantly heterogeneous across different tumor regions and among molecular subtypes (57). Medulloblastomas are classified into Wingless (WNT), Sonic Hedgehog (SHH), Group 3, and Group 4 molecular subtypes, each with a distinct tumor microenvironment and vascular architecture. Notably, the SHH subtype displays specific vascular biological features and differs in the BBB morphology and permeability from the other subtypes (Table 1) (58). The SHH and Group 4 subtypes of medulloblastoma exhibit an intact BBB, whereas the Group 3 subtype shows mild BBB disruption. In contrast, in WNT-activated medulloblastoma, the BBB is notably leaky (Figure 1) (33, 59). Phoenix et al. demonstrated in genetically engineered mouse models that medulloblastomas driven by alterations in the WNT signaling pathway exhibited a compromised BBB, resulting in greater exposure to systemically administered chemotherapeutic agents and an enhanced tumor response (60). The WNT subgroup has an innately porous BBB, which is driven by abnormal β-catenin signaling and causes a fenestrated vasculature (60, 61). This intrinsic property likely enables a chemotherapy drug to pass into the tumor more easily. In contrast, medulloblastomas that are associated with alterations in the SHH pathway tend to maintain a more intact BBB, which renders them less responsive to chemotherapy (60). The SHH subtype also has upregulated angiogenesis pathway molecules, including chemokine receptor type 4 (CXCR4) and vascular endothelial growth factor (VEGF) (62, 63). Research conducted by Tylawsky DE et al. revealed that P-selectin-targeted nanocarriers induced active crossing of the blood‒brain barrier via caveolin-1-dependent transcytosis in the SHH subtype (64), demonstrating that even tumors with intact BBBs (e.g., SHH subtype) may be susceptible to targeted transcytosis-based drug delivery strategies. These preclinical findings align with clinical observations, as patients with WNT-driven medulloblastoma typically exhibit superior survival outcomes compared to those with SHH-driven tumors under similar treatment regimens. While enhanced BBB permeability in the WNT subtype may contribute to improved drug delivery, other contributing factors such as a favorable immune microenvironment and inherent chemosensitivity are also believed to influence the improved prognosis observed in patients (65). Compared with the other subgroups, the Group 3 subtype has significantly greater vascular endothelial growth factor A (VEGFA) mRNA expression (66), which may be directly driven by MYC activation (67). Although some degree of BBB disruption is observed in medulloblastomas, most tumors retain partial barrier function, which poses a major obstacle for systemic therapies to effectively penetrate into the tumor core (57). Group 3 subtype is associated with a poor prognosis, characterized by a survival rate below 40% and frequent metastases (68). Medulloblastoma frequently metastasizes through cerebrospinal fluid (CSF) dissemination, with the therapeutic challenge being especially pronounced in patients presenting with leptomeningeal spread or distant metastases. In these cases, the BBB remains a significant obstacle to effective drug delivery. Intrathecal administration offers considerable potential as a therapeutic strategy to bypass the BBB and directly target tumor cells within the CSF compartment (69, 70). The interaction between tumor cells and the vascular microenvironment also plays a crucial role in maintaining BBB stability. Some studies have shown that SOX2-positive medulloblastoma stem-like cells tightly envelop capillaries via a mechanosensitive ion channel Piezo2-dependent mechanism, thereby influencing local tissue mechanical properties and vascular permeability (71, 72). Targeting the Piezo2 channel (e.g., via knockout) not only significantly enhances drug delivery efficiency but also has the potential to prolong survival, indicating that Piezo2 is a novel target with clinical translational potential (72). Furthermore, radiotherapy has been demonstrated to improve the efficiency of fucoidan nanocarrier-mediated transcytosis via P-selectin, thus increasing drug delivery (64). However, radiation also impacts BBB-associated structures, such as the perivascular space (PVS), potentially causing side effects, including cerebral edema and alterations in the tumor microenvironment (73). Owing to the barrier effect of the BBB, liquid biopsy markers such as circulating microRNAs in peripheral blood have shown limited sensitivity for detecting medulloblastoma (74). Therefore, the clinical focus has increasingly shifted toward the use of imaging parameters to evaluate the BBB status. MRI-based biomarkers, such as the ADC, hold promise for noninvasively monitoring BBB integrity within tumors and assessing treatment responses (75, 76).

**Table 1:** BBB and vascular features in medulloblastoma subtypes.

Subtype	BBB status	Key features	Drug response
SHH	Intact	Low permeability Elevated expression of CXCR4 and VEGF P-selectin–mediated transcytosis	Poor penetration; Lower response
WNT	Leaky	Fenestrated vasculature Abnormal β-catenin–driven permeability	High drug penetration; Good response
Group 3	Compromised	Overexpression of VEGFA	Variable response
Group 4	Intact	Less characterized vasculature	Limited drug delivery

BBB, blood–brain barrier; SHH, sonic hedgehog; WNT, wingless.

## Ependymoma

Ependymoma is a neuroepithelial tumor that arises from the ependymal layer bordering the cerebral ventricles and spinal canal. Intracranial ependymoma represents a major encephalic tumor in children, whereas spinal ependymoma develops more frequently in adults (77). In pediatric ependymomas, the structural integrity of the BBB remains preserved (Figure 1) (78). However, ependymal cells constitute a key component of both the cerebrospinal fluid–brain barrier and the blood–CSF barrier, playing a vital role in maintaining CNS homeostasis. During ependymoma development, these barrier cells can be directly disrupted by the tumor, leading to abnormal infiltration of cerebrospinal fluid components into the tumor parenchyma, which alters the local microenvironment and may promote tumor progression (79). Animal model studies further demonstrated that intracranial tumor growth could directly damage the ependymal epithelial layer, disturbing its barrier function and thereby enhancing interactions between cerebrospinal fluid and tumor tissue (80). Significant regional heterogeneity of the BBB has been observed in ependymomas, particularly in supratentorial tumors. In 9 out of 20 cases, tumor cells in supratentorial ependymomas highly expressed the tight junction protein claudin-5, a marker of BBB integrity, a feature not observed in infratentorial tumors (81). The heterogeneity of the BBB in ependymomas is a major contributor to treatment failure and poor drug delivery efficiency. Targeted therapies face clinical challenges, partly because of the variable drug permeability across different tumor regions, which is governed mainly by differences in BBB permeability and molecular tumor characteristics (82). Some studies have shown that the regulation of BBB functionality is closely linked to the glycosylation of certain transmembrane proteins, such as basigin, which is upregulated in ependymomas. Basigin may influence barrier permeability by modulating endothelial tight junctions and transport mechanisms (83). Ginguené C et al. reported that a biochemical, transporter-dependent blood‒tumor barrier might exist in ependymomas, which may reduce the tumoral bioavailability of lipophilic and amphiphilic anticancer drugs (77). It is noteworthy that two pediatric cases of relapsing demyelination, occurring after and in conjunction with radiation therapy for ependymoma, have been reported with features consistent with a multiple sclerosis phenotype, suggesting radiation-induced disruption of the BBB (84). Such BBB impairment may enhance the permeability of therapeutic agents, potentially improving drug delivery to the central nervous system. In parallel, Ependymal cells regulate CSF dynamics and contribute to CSF production via the choroid plexus. Their strategic ventricular location enables broad distribution of therapeutic proteins through CSF pathways, potentially bypassing the BBB and offering a route for targeted brain tumor treatment while minimizing neuronal toxicity (85). Therefore, a deeper understanding of BBB functional states and underlying molecular mechanisms across ependymoma is critical for improving drug delivery strategies and enhancing therapeutic responses.

## Craniopharyngioma

Craniopharyngioma is a benign tumor originating from embryonic remnants of Rathke's pouch or the residual craniopharyngeal duct epithelium. In pediatric patients, the peak incidence of craniopharyngioma occurs between 5 and 15 years of age, with the adamantinomatous subtype being the most predominant form observed in this population. Pediatric adamantinomatous craniopharyngiomas (ACPs) predominantly occur in the sellar and suprasellar regions, adjacent to critical neurovascular structures such as the hypothalamic‒pituitar*y* axis and optic pathways (86). The integrity of the BBB in this area is crucial for maintaining neuroendocrine system homeostasis. Craniopharyngiomas, though histologically benign, can exert significant mass effects that compress or infiltrate adjacent brain structures, leading to focal BBB disruption (Figure 1) (57, 87). For instance, hypothalamic damage frequently results in multiple pituitary hormone deficiencies and may induce metabolic disorders such as nonalcoholic fatty liver disease, suggesting that BBB dysfunction may play a significant role in these pathological changes (88, 89). Moreover, the tumors' clinically aggressive behavior may further impair BBB integrity, causing neuroendocrine abnormalities (e.g., growth hormone deficiency or diabetes insipidus) as well as visual impairment (90, 91). Studies indicate that pediatric patients face a significantly greater risk of postoperative obesity, cognitive decline, and cranial nerve damage than adults do, which is potentially related to inflammation or oxidative stress triggered by BBB disruption (92, 93). A small phase 0 study, which involved three child patients, provided compelling evidence that the humanized monoclonal antibody tocilizumab effectively reached ACP tumors and cyst fluid after systemic delivery (94). These findings support existing data that indicate that tocilizumab may play a role in systemic therapy for ACP. Furthermore, these findings imply that the ACP-associated BBB is compromised relative to other regions of the CNS (94). Moreover, abnormal expression of tight junction proteins such as claudin-5 within the gut–brain axis is thought to exacerbate BBB dysfunction, suggesting that the peripheral system may influence central nervous system function through the modulation of barrier integrity (95). Currently, surgical resection and radiotherapy remain the primary treatment modalities in patients with craniopharyngiomas but may exacerbate BBB damage to some extent. For example, perioperative BBB disruption is closely associated with cognitive impairment and increased postoperative mortality (93). Oxytocin treatment of ACP patients can restore the integrity of the BBB and reduce the inflammatory response that is activated by infiltrating peripheral immune cells, thus blocking the lipotoxicity of hypothalamic neural stem cells induced by the diffusion of peripheral low-density lipoprotein cholesterol into the third ventricle (96). To minimize hypothalamic injury, some studies recommend conservative treatment strategies, such as limited resection combined with moderate radiotherapy (97, 98). Additionally, the permeability of the BBB in ACP can vary considerably, resulting in inconsistent therapeutic delivery (99). To address this challenge, localized treatment strategies such as intracystic drug administration and nanotechnology-based delivery systems are being investigated as potential approaches to bypass the BBB and improve treatment efficacy (100). However, there is a lack of sufficient evidence regarding the long-term efficacy of these approaches in protecting or restoring BBB function, highlighting the need for further systematic investigations.

## Conclusion

The review underscores the heterogeneous integrity of the BBB across pediatric brain tumors and its significant implications for drug delivery and therapeutic resistance. By integrating histopathological, molecular, and imaging data, it highlights the subtype-specific characteristics of BBB disruption and preservation. Furthermore, the review evaluates emerging therapeutic strategies, such as FUS and CED, that aim to overcome BBB-associated barriers, thereby enhancing drug delivery and potentially improving clinical outcomes in affected children.
